# FRASS: the web-server for RNA structural comparison

**DOI:** 10.1186/1471-2105-11-327

**Published:** 2010-06-16

**Authors:** Svetlana Kirillova, Silvio CE Tosatto, Oliviero Carugo

**Affiliations:** 1 Department of Structural and Computational Biology, Max F Perutz Laboratories, Vienna University, Campus Vienna Biocenter 5, A-1030 Vienna, Austria; 2 Department of Biology, Universita' di Padova, Viale G Colombo 3, I-35121 Padova, Italy; 3 Department of General Chemistry, Pavia University, Viele Taramelli 12, I-27100 Pavia, Italy

## Abstract

**Background:**

The impressive increase of novel RNA structures, during the past few years, demands automated methods for structure comparison. While many algorithms handle only small motifs, few techniques, developed in recent years, (ARTS, DIAL, SARA, SARSA, and LaJolla) are available for the structural comparison of large and intact RNA molecules.

**Results:**

The FRASS web-server represents a RNA chain with its Gauss integrals and allows one to compare structures of RNA chains and to find similar entries in a database derived from the Protein Data Bank. We observed that FRASS scores correlate well with the ARTS and LaJolla similarity scores. Moreover, the-web server can also reproduce satisfactorily the DARTS classification of RNA 3D structures and the classification of the SCOR functions that was obtained by the SARA method.

**Conclusions:**

The FRASS web-server can be easily used to detect relationships among RNA molecules and to scan efficiently the rapidly enlarging structural databases.

## Background

Three-dimensional (3D) structures of RNA have been extensively studied because of the importance of these molecules that are involved into several biological processes. Structural analysis is also essential for molecular evolution investigations and the development of structure prediction tools.

The flexibility of the RNA backbone, limited to six torsion angles per base, is restricted. It was shown [1-3] that RNA molecules can be represented by using collections of stable structures or structural motifs. Therefore, most of the 3D tools developed for RNA molecules handle only local structural elements or motifs.

PRIMOS [4] uses the 3D worm representation of a RNA molecule in which two coordinates correspond to the pseudo-torsions describing the nucleotide conformation and a third coordinate defines the sequence. PRIMOS was used to perform structural motif searches and is limited to the comparison of different conformations of the same molecule. COMPADRES [5] is a modified version of PRIMOS, developed to discover new motifs. The NASSAN method [6] uses the graph representation of a RNA structure and applies the Ullmann subgraph isomorphism algorithm [7] for structural comparison. The efficiency of the Ullmann algorithm rapidly decrease with the number of nodes in the query motif (subgraph) and it is not suitable for large molecules. In the method proposed by Apostolico et al. [8], RNA motifs are represented by the histograms of the distances between all backbone atoms and the centroid of the phosphate atoms. Thus, 3D comparisons are reduced to comparisons of the shapes of the histograms. The FR3D [9] method was also developed for finding local motifs in RNA structures. It applies the base centered approach in which each base is represented by the position of its glycosidic nitrogen in 3D space and by the rotation matrix that allows its orientation with respect to a common frame. The automated approach for identification of RNA conformational motifs proposed by Hershkovitz et al. [10] is based on binning of the values of the torsion angles defining the nucleotide conformation. This method makes possible to use alphabetic descriptions of 3D structures. It was later extended, by the RNA Ontology Consortium, into the "consensus modular string nomenclature" for RNA structures, which includes 46 discrete conformers represented by two characters [11]. Although the techniques described above were successfully applied for comparison, searching, and discovering of structural motifs, a unique, universal, and effective method for RNA structural comparison is still lacking. Thus, the well-known classification of RNA structural motifs - the SCOR database - was carried out manually [12]. This fact can be explained by the irregularity of the RNA 3D structures and discrepancies in the structural motifs definitions [1].

The structural analysis of large and intact RNA molecules, and not only of small fragments, became increasingly more common during the last few years [1,2]. Therefore, methods like DIAL [13], ARTS [14], SARSA [15], SARA [16], and LaJolla [17], suitable for comparison of large global folds, are of key importance. DIAL (dihedral alignment) defines dihedral/pseudo-dihedral similarity using six backbone torsions and includes also nucleotide sequence and base-pairing similarities. The ARTS (alignment of RNA tertiary structure) method produces the score consisting of two components that correspond to the numbers of spatially close base pairs and of single nucleotides. The method was applied to create the DARTS database that is a RNA classification mainly based on global spatial resemblance [18]. SARA produces the structure alignment using unit-vector strategy [19] and estimates the similarity degree of sequence, secondary structure and 3D structure [16]. The SARA web-server provides functional annotations according to the SCOR classification [12]. The SARSA [15] and LaJolla [17] algorithms transform 3D structures into 1D strings of characters that can be aligned and compared with faster techniques.

In the past years the number and size of novel RNA structures has dramatically increased. However, comparison methods based on structural alignments are not sufficiently rapid for interactive scanning of large databases. Quite a few alternative algorithms, which do not produce structural alignments, were designed for rapid comparison of protein structures [20-27]. They are effective for scanning large databases and also provide a quantitative measure of fold similarity to visualize the structural diversity of proteins on 2D or 3D maps using Principal Component Analysis (PCA) [20,21]. One of these structural comparison methods is the Gauss-integrals representation of a protein structure [21-23]. The structure is represented only by positions of backbone C_α _atoms and, therefore, can be applied also to RNA molecules, with minor modifications.

In this article, we describe a web-server developed for global comparison of RNA structures using Gauss-integrals [24]. The FRASS web-server is free and open to all users and there is no login requirement. The server allows both pair-wise comparisons between two structures provided by the user and database scanning, in which the user wants to extract database entries similar to his query. The correlation between the Gauss-integrals based distances and the ARTS and LaJolla similarity scores was observed to be considerable. Moreover, by considering as a benchmark the DARTS and SCOR classifications of RNA structures, it was proven that the method based on the Gauss-integrals allows one to automatically build satisfactory classifications.

## Implementation

## Methods

In the Gauss-integrals approach [21-23], the RNA backbone is regarded as a polygonal space curve *μ *that is a series of connected line segments. The segments are specified by a sequence of phosphorous atoms (P_1_, P_2_, .... P_N_). The first segment is the section between P_1 _and P_2 _atoms, the second segment is the section between P_2 _and P_3_, ect. The shape of the polygonal space curve is described by a 30-dimensional vector containing the length of the backbone and the 29 Gauss integrals of the first, second and third order.

The Gauss-integral of the first order for the RNA molecule containing *N *phosphorous atoms is

where *W(i*_*1*_*,i*_*2*_*) *is the probability of seeing the *i*_*1*_th and i_2_th line segments to cross when averaged over all directions in space and the sign of this crossing is determined by right-hand rule [22]. The Gauss-integral of the second order is

The Gauss-integral of the third order is

The Gauss Integrals are defined by mutual spatial superposition of the segments and represent the mathematical description of the backbone shape of a RNA molecule. The method can not be used for small molecules because the third-order Gauss-integral defined by the spatial superposition of six segments and can be computed only for molecules containing more than 7 nucleotides. 2,703 RNA structures out of the 3,353 deposited into the PDB contain more than 7 nucleotides. The distance between two RNA structures was measured by the Euclidean distance between the two 30-dimensional vectors, one for each RNA molecule.

The statistical significance of the Gauss-integral based distance (D) was determined empirically, by using the distribution of the distances d(x) computed for all pairs of RNA chains of the database. With these data, it is possible to estimate the probability pD to have D values higher than a given value D_x_, simply by integrating the probability density curve from D_x _to D_max_:

### Web server

The RNA structures were downloaded from the Protein Data Bank. Each monomeric RNA molecule was stored in a single PDB-formatted file and its Gauss integrals were computed. Given that the Protein Data Bank is frequently updated, a program for rapidly updating the RNA structure database was developed. The GI program for Gauss-integral calculations is freely available at [25]. In our modified algorithm, the RNA phosphorous atoms are used for backbone description. The program that allows one to scan the RNA structural database and to find the entries that are the most similar to the query was also written locally. By definition, the Gauss-integrals can be used to describe a single polymer chain. Presently, the web-served can process only single chains, though it must be considered that a significant percentage of RNA structures are formed by more than one chain. In this case, each chain should be processed separately.

**Figure 1:**
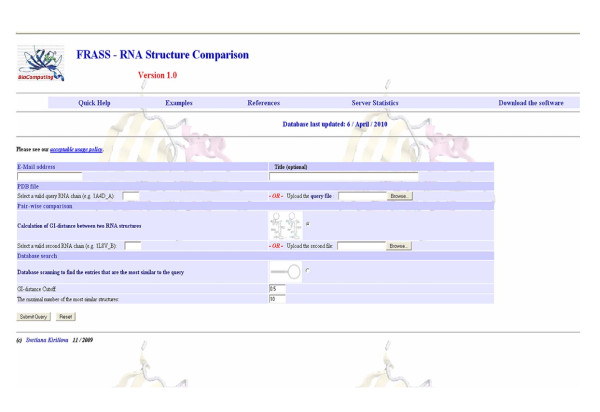
**The input form for FRASS server**. It provides two possibilities to perform structural comparisons: on the one hand, the pair-wise comparison and, on the other, the database scan. The input files must be PDB-formatted. For database scanning, the cut-off distance and the number of similar structures can be specified (though reasonable default values are provided).

**Figure 2:**
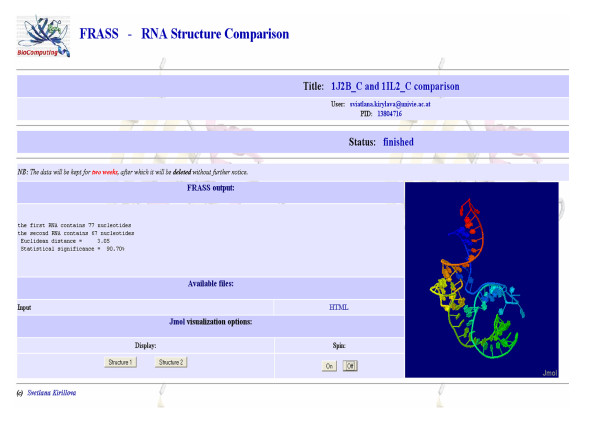
**Sample output of the FRASS server for the pair-wise comparison**. The result of the pair-wise comparison of two tRNA molecules with IDs 1J2B (chain C) and 1IL2 (chain C).

**Figure 3:**
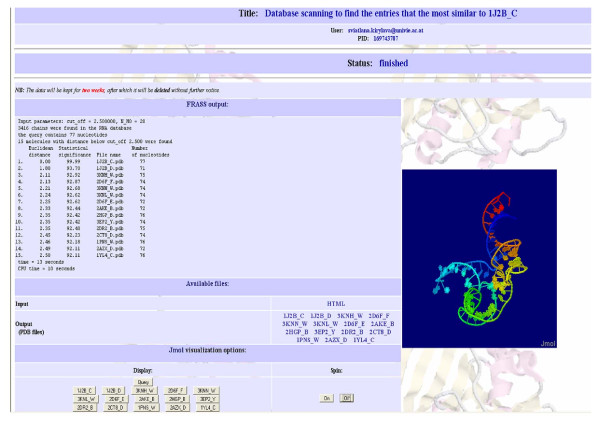
**Sample output of the FRASS server for the database search**. The most similar structures with distance below cut-off 2.5 found for RNA 1J2B (chain C).

The input form for the web server, referred to as FRASS, is shown in Figure 1. It requires the email address of the user and the (optional) title of the job. The user can perform the pair-wise comparison of two RNA structures or scan the database of RNA structures to find entries similar to the query. In the case of the pair-wise comparison, the two PDB files specified as "the query file" and "the second file" must be uploaded. If the PDB file contains more than one chain/model, the first chain/model will be considered. In the case of database scanning, only the query file must be uploaded. In this case the user can define two parameters: the Gauss-integrals distance cut-off and the number of the most similar structures which will be retained from the RNA database. The default values of these parameters are 0.5 and 10, respectively. Sample outputs of the FRASS server are shown in Figure 2 and Figure 3. The output of the pair-wise comparison includes the Gauss-integrals distance between the two structures (Figure 2). For example, the Gauss-integrals distance between two tRNA molecules with PDB identification codes 1J2B (chain C) and 1IL2 (chain C) is equal to 3.05. To estimate the statistical significance of this value, it was necessary to compute the distribution of the distances obtained for all pairs of RNA molecules of the database and in this case 90.70% of all the distances are larger than 3.05. This implies that only less than 10% of them are smaller and it suggests that this level of similarity is rather uncommon. The output of the database scanning is the list of the entries that are more similar than the cut-off distance. The RNA structure 1J2B (chain C) was chosen to exemplify the FRASS output (Figure 3). 1J2B is a tRNA with lambda-form that drastically differs from the typical tRNA L-shape. Whereas 141 tRNA structures are in one DARTS cluster, the chain C of 1J2B was registered as a false negative in the classification [18]. Most of the alignments of the C chain of 1J2B with other tRNA chains made with the LaJolla method result in a small number of pair-wise superpositions of equivalent nucleotides [17]. A multiple global structural alignment of the chain C of 1J2B with five tRNA chains (H4S_T, 1ASZ_R:620-660, 1IL2_C, 2CSX_C, 1EVV_A), created with the SARSA method, resulted in a large RMSD of 10.73 Å [15]. By using the chain C of 1J2B as a query in the FRASS database search mode (and by using a cut-off distance of 2.5 and by limiting to no more than 20 the number of database entries to be shown in the output), 15 tRNA chains were retained from the database (Figure 3). Their distances from the query are significantly small, since more than 92% of the possible distances are larger than them. This indicates, that the Gauss-integrals based method implemented into the FRASS server is able to successfully handle the difficult case of a tRNA chain (chain C of 1J2B) that is categorized into a cluster different from the cluster of other tRNAs in the DARTS classification.

## Results

### Comparison with ARTS and LaJolla similarity scores

**Figure 4:**
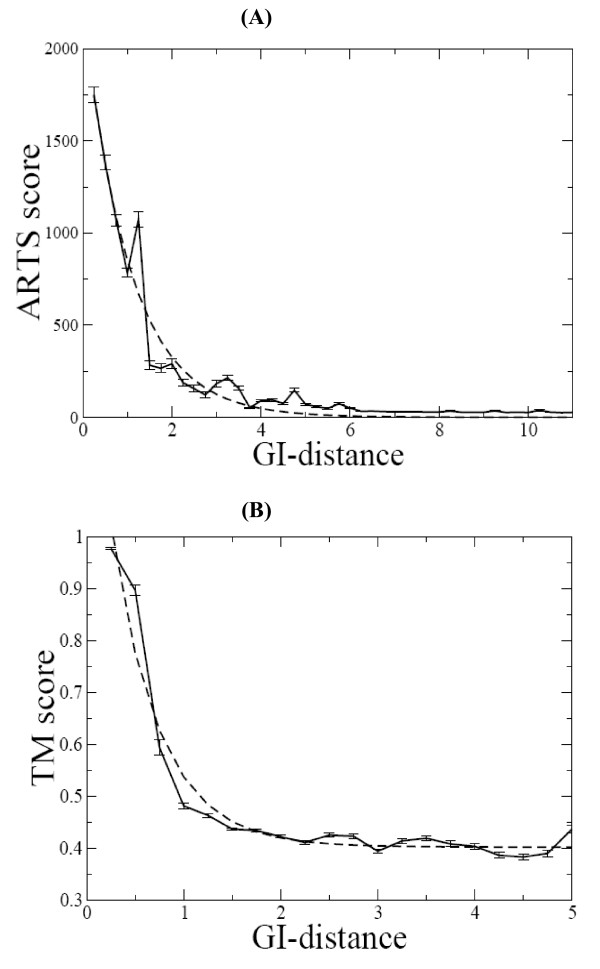
**Relationship between Gauss-integrals distances and ARTS and LaJolla scores**. (**A**) Average values of the ARTS scores (with standard deviations as vertical bars) as a function of Gauss-integrals distance. The calculations were carried out on the 820,600 pairs of RNA chains. (**B**) Average values of the LaJolla TM scores (with standard deviations as vertical bars) as a function of Gauss-integrals distance. The calculations were carried out on the 5,050 pairs of tRNA chains. The dashed lines represent non-linear exponential curve fitting performed by GRACE program [30] with correlation coefficient -0.98.

The Gauss-integrals based algorithm was benchmarked against the ARTS method by using all RNA structures that were suitable for ARTS, which does not process single chain molecules that lack base pairs. The correlation between the Gauss-integrals based distances and the ARTS similarity scores was analyzed. Figure 4A shows the relationship between the ARTS scores and the Gauss-integrals Euclidean distances. This relationship, determined on the basis of 820,600 pairs of RNA chains, can be fitted by the exponential curve

The FRASS algorithm was benchmarked also against the LaJolla method, using the dataset of 101 tRNA chains reported in [17] that implies 5,050 unique pairs of RNA structures. Figure 4B shows the relationship between the similarity scores (named TM) produced by LaJolla and the Gauss-integrals Euclidean distances, which can also be fitted by an exponential curve

Pearson correlation coefficients are equal to -0.98 in both cases. The correlations are negative since the Gauss-integrals based scores are distances while the ARTS and LaJolla scores are similarities.

### Benchmarking against the DARTS and SCOR classifications

An effective way to test the classification ability of a method is to compute a ROC curve. In the present study, the DARTS and SCOR classifications of RNA structures was used as an external benchmark. The database DARTS contains 1,333 RNA structures determined experimentally. They are classified into 94 clusters. Only the 789 single chain entries of DARTS were retained, since the web server described in the present manuscript handles only monomeric molecules. Gauss-integrals based distances were thus computed for 310,866 pairs of RNA structures. The NR95-SCOR dataset available at [26] contains 60 RNA chains that have more than 20 and less 300 nucleotides and are assigned to SCOR functional classes with SARA. This results in 1,770 unique pairs of RNA structures.

The ROC curve is obtained by plotting *Sensitivity *against *(1-Specificity) *defined as

where true positive (TP) and false positive (FP) that are the number of correctly and incorrectly predicted pairs of the same DARTS/SCOR cluster, while true negative (TN) and false negative (FN) are the number of correctly and incorrectly predicted pairs of different clusters. Different points in the ROC curve are obtained by varying the Gauss-integrals based distance value under which two structures are considered to be similar and to belong to the same DARTS/SCOR cluster.

**Figure 5:**
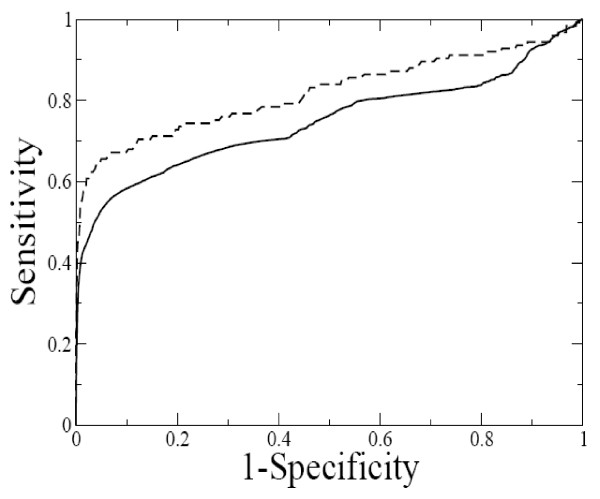
**The ROC curves**. The solid line represents the curve computed for 789 RNA chains taken from the DARTS classification (AUC = 0.75). The dashed line was obtained by using the NR95-SCOR dataset (AUC = 0.86).

Figure 5 shows the ROC curves obtained as described above. The areas under ROC curves (AUC) are 0.75 and 0.82, respectively for the DARTS and SCOR classifications. These values monitor the performance of the method. A value equal to 0.50 would be associated with a random similarity measure, while a value equal to one would be obtained with an impeccable similarity measure. The AUC values obtained in the present study compare well with those obtained with the DIAL [13], SARSA [15], and SARA [16] methods, which range from 0.58 to 0.86 depending on which benchmarking set is used and on the fine tuning of each method.

### Computational time

The computation of the Gauss integrals is O(n^3^) in time and it was observed that for long molecules (~1,500 nt) it takes about half an hour on a standard PC. Therefore, dealing with a large database, the Gauss integrals must be pre-computed and stored on a hard disk. The computing of Euclidean distances between pre-calculated Gauss integrals is on the contrary extremely fast and the database scanning takes very few seconds on a standard PC.

Although the fact that FRASS does not produce structural alignments, it must be observed that, in general, methods that generate structural alignments are not suitable to work with large databases containing long RNA chains: although ARTS is O(n^3^) in time, DIAL is O(n^2^) in time, and LaJolla and SARSA are O(1) in time, nothing can be pre-computed and stored on a hard disk for further elaboration. The SARSA and LaJolla methods, which transform 3D structures into 1D strings, are faster than other techniques. In particular, SARSA was shown to be faster than DIAL, though no quantitative information was published [15]. LaJolla takes about 15 minutes on a standard PC to generate 5,050 alignments of RNA chains (see the datasets described in the paragraph "Comparison with ARTS and LaJolla similarity scores"). On the contrary, the computation of 5,050 Gauss-integrals based distances takes only about one second. Moreover, structural alignments and function assignments with the SARA server are limited to RNA structures with less than 1,000 nucleotides, since computations are very demanding.

### Global similarity of 23S ribosomal RNAs

The large 23S ribosomal RNA from *Haloarcula marismortui*, the crystal structure of which was refined at 2.4 Å resolution [27], was chosen to test the web-server. As a query, we selected the chain 0, containing about 2,700 nucleotides, taken from 1FFK file of the Protein Data Bank. The most similar structure found in the database using the FRASS web-server is the 23S ribosomal RNA from *Deinococcus radiodurans *(PDB identification code 3CF5, chain X, about 2,700 nucleotides) [28]. The Gauss-integrals distance between the two structures is equal to 1.2 that reveals their high structural similarity because 96% of distances computed for all pairs of RNA database are larger than 1.2. The similarity ARTS score equal to 3,294.00 corresponds to 588 aligned base-pairs and 2,118 aligned nucleotides. The high global similarity detected by both methods supports the similar biological activity of the two molecules that was also analyzed in recent, detailed comparisons of their structures and functions [28,29].

## Conclusions

The FRASS web-server is an effective tool for global comparison and classification of RNA structures. The similarity measure, based on the Gauss-integrals, is related to the backbone shape of a single RNA chain, represented by the positions of the phosphorous atoms. It is alternative and complementary to other similarity scores that considers base-pairs. Given the simplification of the backbone representation, computations are extremely fast. The web-server allows thus database scanning that can be used to detect relationships among RNA molecules, and to assign function to a new experimentally determined structure on the base of the structural similarity.

## Availability and requirements

• **Project name**: FRASS

• **Project home page**: http://sourceforge.net/projects/frass/

• **Operating systems**: Platform independent for web-server, Linux for downloaded software

• **Programming languages**: C and Perl

• **License**: GNU GPL

## Authors' contributions

SK developed the tool and drafted the manuscript. SCET and SK elaborated the tools into the web-server. OC initiated and supervised the project. All the authors read and approved the manuscript.
